# A gateway for ion transport on gas bubbles pinned onto solids

**DOI:** 10.1038/s42004-021-00481-7

**Published:** 2021-03-25

**Authors:** Veton Haziri, Tu Pham Tran Nha, Avni Berisha, Jean-François Boily

**Affiliations:** 1 Department of Chemistry, University of Prishtina, Prishtina, Kosovo; 2 Department of Chemistry, Umeå University, Umeå, Sweden

**Keywords:** Scanning probe microscopy, Electrochemistry, Geochemistry

## Abstract

Gas bubbles grown on solids are more than simple vehicles for gas transport. They are charged particles with surfaces populated with exchangeable ions. We here unveil a gateway for alkali metal ion transport between oxygen bubbles and semi-conducting (iron oxide) and conducting (gold) surfaces. This gateway was identified by electrochemical impedance spectroscopy using an ultramicroelectrode in direct contact with bubbles pinned onto these solid surfaces. We show that this gateway is naturally present at open circuit potentials, and that negative electric potentials applied through the solid enhance ion transport. In contrast, positive potentials or contact with an insulator (polytetrafluoroethylene) attenuates transport. We propose that this gateway is generated by overlapping electric double layers of bubbles and surfaces of contrasting (electro)chemical potentials. Knowledge of this ion transfer phenomenon is essential for understanding electric shielding and reaction overpotential caused by bubbles on catalysts. This has especially important ramifications for predicting processes including mineral flotation, microfluidics, pore water geochemistry, and fuel cell technology.

## Abstract (web summary)

Electrocatalytic reactions depend on ion transport phenomena within the electric double layer, but the electrochemical response of single gas bubbles that form on catalyst surfaces remains unclear. Here, the authors use alternating current electrochemical impedance spectroscopy to detect a gateway through which ions can exchange between gas bubble surfaces and solid-state catalyst surfaces.

## Introduction

Gas bubbles are to the human eye the most tangible forms of dissolved gases in water. Their study involves a captivating blend of chemistry and physics, and has a wide range of ramifications to science and technology^1–3^. Solid surfaces can collect gas bubbles by processes including direct immersion of solids in water, temperature or pressure variations, solvent exchange, microwaves, ultrasounds, cosmic rays, and (photo)(electro)chemical gas evolution reactions^1–3^. Knowledge and control of gas bubble formation are strongly needed for technological applications as varied as fuel gas production, mineral separation, medical imaging and green technologies^1,4,5^. This can be especially important for understanding how bubbles impact catalytic reactions, how they block liquid flow in porous networks and, when desired, how they can be eliminated^6–12^.

**Fig. 1:**
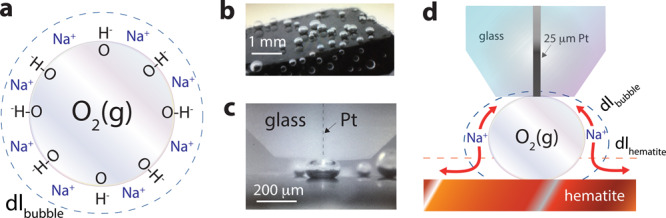
Electrochemically produced and measured O_2_(g) microbubbles. **a** Schematic representation of a single oxygen bubble with its surface OH^−^ groups and Na^+^ counterions, forming an electric double layer (dl_bubble_; region capped by dashed blue line). **b**, **c** Photography of electrochemically produced O_2_(g) bubbles via water oxidation on a single hematite (α−Fe_2_O_3_) electrode, and **c** of an individually probed bubble with an ultramicroelectrode (UME; 25 μm-wide Pt). **d** Schematic representation of alternating current scanning electrochemical microscopy (AC-SECM) measurement of a single O_2_(g) bubble with an UME (25 μm-wide Pt core). Red arrows denote the gateway for ion transport enabled by overlapping electric double layers of the bubble and hematite (dl_hematite_; region capped by dashed red line). See Supplementary Fig. 1 for complete representation of the 3-electrode system used for these measurements and Supplementary Fig. 2 for bubble size distributions.

Gas bubbles are capped by an electric double layer (EDL) resulting from the preferential binding of hydroxide ions at the air/water interface^13–21^ (isoelectric point at pH ~3–4)^22^, and countercations neutralizing this charge (Fig. 1a)^13–17^. The mobility of these ions within the EDL reflects the ohmic conductivity of bubble surfaces, and is thus an important aspect to consider for understanding how pinned bubbles (Fig. 1b, c) can electrically shield solids, block reaction sites, or cause reaction overpotential during catalysis^9,23–25^. At the core of these phenomena lies the possibility for the exchange of ions from the overlapping EDL of bubbles and the solid surfaces upon which they are pinned (Fig. 1d).

Previous studies dedicated to bubble electrochemistry have predominantly involved large populations of bubbles. For instance, atomic force microscopy^26,27^ and scanning electrochemical microscopy^10,28–30^ have been used to map the spatial distributions of bubbles populating catalyst surfaces. Work with microelectrodes also uncovered electrically resistive populations of gas bubbles^29^. No studies have, however, directly focused on the electrochemical response of single gas bubbles, which is needed to resolve ion transport phenomena directly within the EDL.

Using a ultramicroelectrode (UME; Fig. 1c, d), we here directly measured the electrochemical impedance of single oxygen bubbles pinned onto solids. We reveal a gateway through which ions can transfer between the overlapping EDLs of gas bubble and (semi)conducting solid surfaces. Harnessing this gateway could even offer new avenues in the study of gas-evolving reactions of natural and technological importance. In particular, our primary focus on hematite (α-Fe_2_O_3_), as an oxygen evolution reaction catalyst, directly relates to emerging water-splitting technologies^31,32^, and for the important roles it plays in the biogeochemical cycling of elements on Earth, and even on the geochemistry of planet Mars^33–35^.

## Results and discussion

**Fig. 2:**
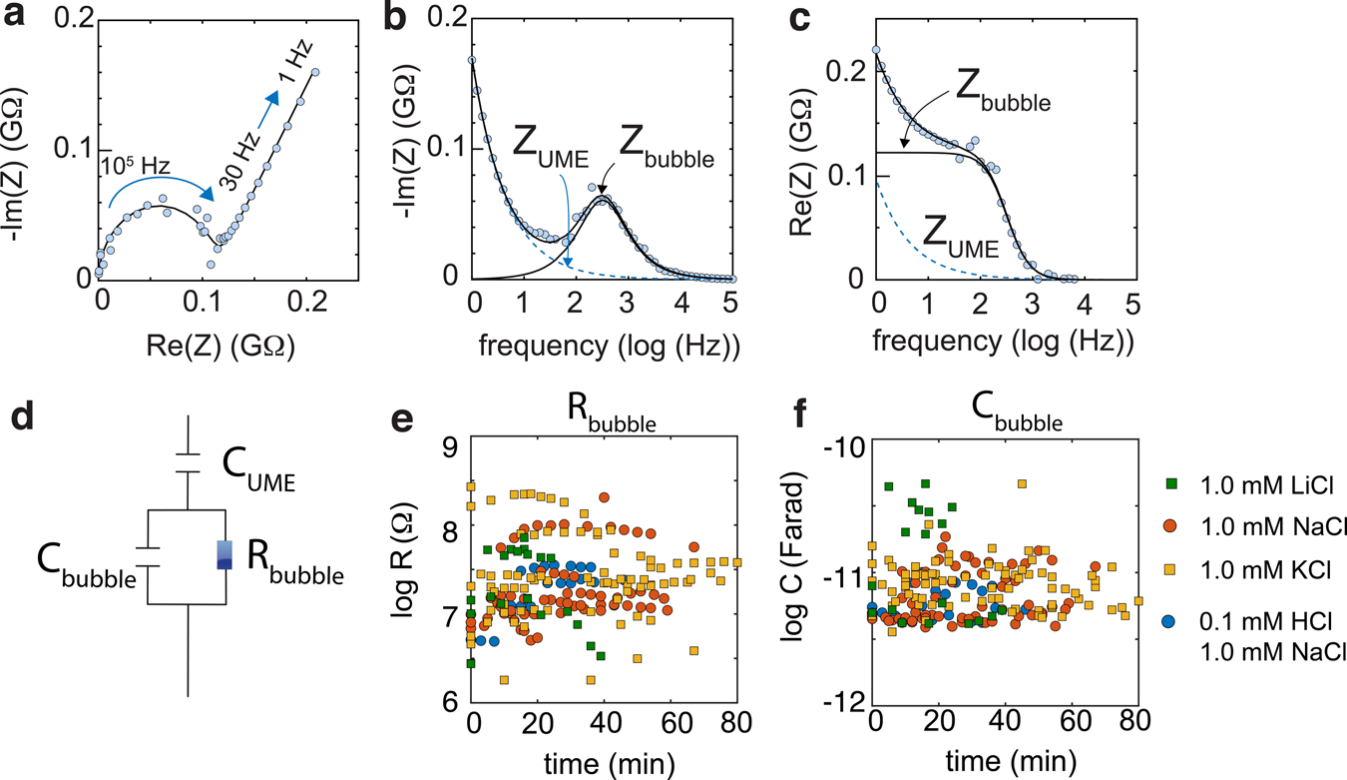
EIS data and model predictions and parameters. **a** Example of a typical Nyquist (complex-plane impedance) plot showing real (Re) and imaginary (Im) impedance (Z) data (blue circles) and model (lines) of an oxygen bubble pinned on hematite in an aqueous solution of 1.0 mM NaCl at 25 °C. Measurements were collected over an applied alternating current of ±50 mV in the 1–10^5^ Hz range through an ultramicroelectrode (UME). **b**, **c** Frequency-resolved **b** imaginary and **c** real impedance data (blue circles) alongside predicted contributions from the bubble and the UME (lines). **d** Equivalent circuit model used to predict bubble polarization resistance (R_bubble_) and double layer capacitance (*C*_bubble_), alongside UME capacitance (*C*_UME_). **e** *R*_bubble_ and **f** *C*_bubble_ values from repeated EIS measurements on 29 different oxygen bubbles pinned on hematite in four different ionic media (green square = 1.0 mM LiCl, red circle = 1.0 mM NaCl, yellow square = 1.0 mM KCl, blue circle = 0.1 mM HCl + 1.0 mM NaCl), and for repeated measurements on single bubbles over a period of up to 80 min. Supplementary Figs. 3–4 contain the complete set of model parameters and distributions of these values. Supplementary Fig. 5 shows similar ranges of model parameter values for bubbles pinned on gold.

The impedance (*Z*) of submillimeter-sized (~144–460 μm; Supplementary Fig. 2) oxygen bubbles pinned on hematite were measured with a UME by Electrochemical Impedance Spectroscopy (EIS)^36^ (Figs. 1c, d and 2a). EIS measurements over a range of alternating current (AC) frequencies (Fig. 2a–c) revealed charge carrier transport processes at bubble surfaces. Typical complex-plane impedance data (Fig. 2a) revealed a low frequency response below ~30 Hz from the UME, and of a semi-circle in the ~30–3000 Hz region. This latter region was modeled with an equivalent circuit model comprised of a parallel combination of a polarization resistance and of double-layer capacitance (Fig. 2d). The absence of response at frequencies above ~3000 Hz shows that the applied AC cannot trigger space charge or bulk electron transport in hematite, as we have previously reported when the UME is in direct electrochemical contact with hematite^37–40^. As the ~30–3000 Hz region is more typical of solution-side processes^37,38^, we assigned the polarization resistance (*R*_bubble_) and double layer capacitance (*C*_bubble_) terms of the equivalent model to ion transport at bubble surfaces.

To explore variations in the electrochemical response of bubble surfaces, we performed EIS measurement on 29 different bubbles, each in a set of repeated measurements over the course of up to ~80 min. These measurements revealed a spread of *R*_bubble_ values within the ~0.1–1 MΩ range and of *C*_bubble_ within the 5–10 pF·s^−φ^ range (Fig. 2e, f, Supplementary Figs. 3–4). These values were independent of bubble diameter (Supplementary Fig. 2), and were within the same range as those obtained for bubbles pinned on gold (Supplementary Fig. 5). Measurements in solutions of different background electrolytes (LiCl, NaCl, HCl+NaCl, KCl) also revealed that contrasting solution conductivities were not sufficiently important to be manifested within the range of *R*_bubble_ and *C*_bubble_ values (Fig. 2e, f).

To evaluate whether the span in *R*_bubble_ and *C*_bubble_ values arose from a heterogenous response from the bubble surface (e.g., variations in roughness, composition, or non-uniform current distribution)^41^, we examined the breadth of the frequency-resolved distribution of the impedance data (Fig. 2b, c). This was achieved by evaluating the non-ideality factor (*φ*) of the constant phase element (CPE) of our equivalent circuit model (Fig. 2d; Eq. 3)^36,41^. As the resulting values were close to unity (*φ* = 0.9–1.0) on both hematite (Supplementary Fig. 2) and on gold (Supplementary Fig. 4), the response of the bubble surfaces was predominantly homogeneous. A possible source for the spread in response could, on the other hand, have arisen from variations in the contact area in a gateway for ion transport between the bubble and the supporting solids. These could be caused by physical irregularities of the substrate, such as cracks and cavities^42^.

**Fig. 3:**
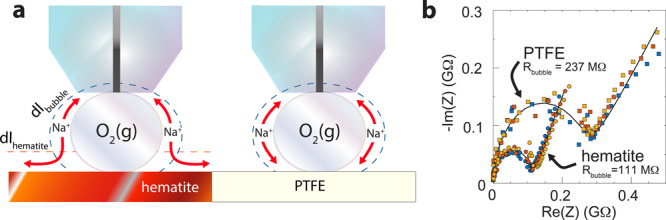
EIS response of bubbles pinned on semi-conducting and insulating surfaces. **a** Schematic representation of sequential EIS measurements of a single oxygen bubble, first on semi-conducting hematite then after displacement on an adjacent insulating PTFE support. **b** Nyquist (complex-plane impedance) plot showing real (Re) and imaginary (Im) impedance (Z) data (triplicates denoted by colored circles and squares) and model (lines) of an oxygen bubble pinned on hematite (circles) and PTFE (squares) in an aqueous solution of 1.0 mM NaCl at 25 °C. Measurements were collected over an applied alternating current of ±50 mV in the 1–10^5^ Hz range. Solid lines are fits from the equivalent circuit model of Fig. 2d, and result from contrasting bubble resistance values (*R*_bubble_). More results are in Supplementary Fig. 6.

To evaluate whether this gateway was affected by the EDL composition of the supporting solid, we compared the impedance response of bubbles pinned on hematite and gold with that of polytetrafluoroethylene (PTFE), an insulator of low wettability (Fig. 3). This was achieved by first repeatedly measuring the electrochemical impedance of a bubble on hematite (Fig. 3, Supplementary Fig. 6) or gold (Supplementary Fig. 6), then displacing the same bubble laterally by 3–5 mm onto an adjacent PTFE support where additional impedance data were collected. We find that this displacement had only a marginal impact on double layer capacitance, yet it readily increased polarization resistance (Fig. 3). These comparable double layer capacitance values align with the concept that both PTFE and the air/water interface are populated by hydroxide ions and counteractions, and have similar isoelectric points^20,21^. The hike in resistance reveals, on the other hand, that ion transport between the two compositionally similar bubble and PTFE surfaces is inhibited. This situation contrasts starkly with the case of bubbles pinned on hematite and gold where more important differences in double layer capacitance and ionic composition facilitate the gateway for ion transport, as manifested by considerably lower resistances than on PTFE.

**Fig. 4:**
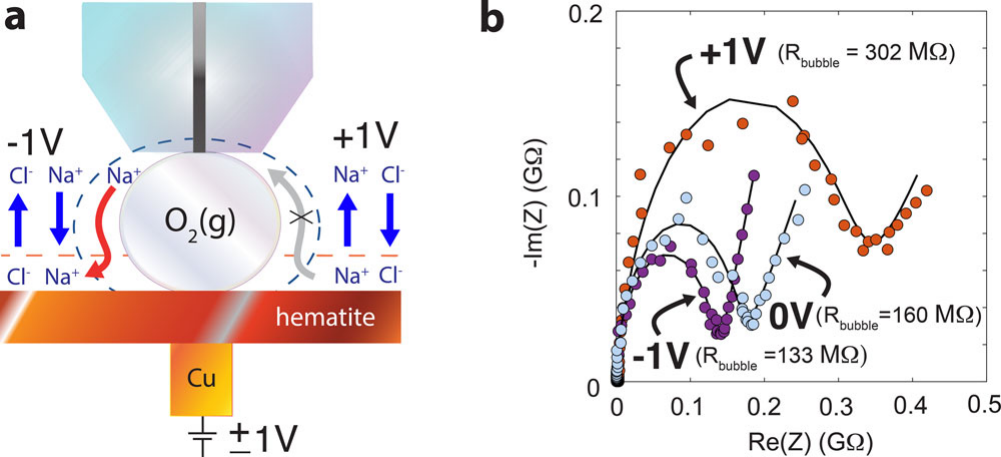
EIS response of bubbles under an externally applied potential. **a** Schematic representation of counterion transport under externally applied potentials of −1 V or + 1 V, here through hematite. The gateway for ion transport is enabled by the overlapping electric double layers of the bubble (capped by dashed blue line) and hematite (capped by dashed red line) at −1 V (red arrow). This gateway is blocked at +1 V (gray arrow with ‘x’). Blue vertical arrows denote direct solution/hematite exchange of counterions affected by the applied potential. While we expect that chloride should bind to hematite, it exerted no response in the EIS data as it has no affinity for the bubble surface. **b** Nyquist (complex-plane impedance) plot showing real (Re) and imaginary (Im) impedance (Z) data (colored circles) and model (lines) of an oxygen bubble pinned on hematite under applied fields of −1 V (purple circles), 0 V (open circuit potential; turquoise circles) and +1 V (red circles). Measurements were collected over an applied alternating current of ±50 mV in the 1–10^5^ Hz range through the ultramicroelectrode, and in an aqueous solution of 1.0 mM NaCl at 25 °C. Solid lines are fits from the equivalent circuit model of Fig. 2d. More results are in Supplementary Fig. 7.

Finally, to confirm further the existence of this gateway, we measured the electrochemical response of the bubble surface with an electric potential applied directly through the supporting solid (Fig. 4, Supplementary Fig. 7). In both hematite (Fig. 4, Supplementary Fig. 7) and gold (Supplementary Fig. 7), we find that bubbles became less resistive (e.g., *~*20% less on hematite) under negative potential of −1 V, but even more resistive than on PTFE under a potential of +1 V (Fig. 4b, Supplementary Fig. 7). We explain our findings based on the attraction and mobility of ions in the EDL. A negative potential draws countercations from the bubble to the supporting solid, explaining the lower resistivity of the bubble. In contrast, a positive potential inhibits countercation transport to the solid, and thus increases bubble resistivity. Also, while a positive potential promotes counteranion binding to the solid, no transport is possible to the negatively-charged bubble. These EIS measurements under externally applied potentials consequently showed that variations in the EDL composition of the supporting surface have a direct impact on bubble impedance, and therefore confirm further the existence of a gateway for ion transport.

## Conclusions

We reveal here a gateway for ion transport between oxygen bubble surfaces and supporting solids of contrasting EDL composition. We represent this gateway in terms of a parallel combination of a constant double-layer capacitance, and of more variable polarization resistance, relating ion transport at bubble surfaces^13–17,39,43^. Variable contact area between bubbles and the supporting solid may be a likely source for variations in bubble impedance. Although active at open circuit potential, direct control of this gateway is also possible by applying an external electric potential passing through the supporting solid. Negative electric potentials facilitate, while positive potentials inhibit, cation exchange between bubble surfaces and the supporting (semi)conducting solid.

By focusing this work on bubbles pinned on semi-conducting hematite, we here resolved a gateway of especially direct relevance to applications including photoelectrochemical water splitting, mineral separation and to redox (geo)chemistry^31–33,44^. Still, the concept of this gateway is highly applicable to a wider range of systems involving gas bubble generation and reactivity at many other (semi)conducting solid surfaces, and even through porous networks within materials of direct relevance to groundwater geochemistry^45^, microfluidics^46^, and fluidized bed reactors^47^.

## Methods

### Oxygen bubble production

Oxygen bubbles were electrochemically produced by water oxidation^48^ using a 2-electrode set-up consisting of hematite (α-Fe_2_O_3_; 5 mm × 5 mm × 3 mm from SurfaceNet) or gold (5 mm circular from BioLogic) working electrodes, and a Pt counter electrode. These reactions were achieved in aqueous solutions of air-saturated 1.0 mM LiCl, 1.0 mM NaCl, 1.0 mM KCl and in a mixed solution of 0.1 mM HCl+1.0 mM NaCl. All electrode surfaces were cleaned prior each experiment for organic impurities by sonication in propanol, followed by ethanol, then in ultrapure water (18.2 MΩ·cm). The working electrodes were mounted at the bottom of a polytetrafluoroethylene (PTFE) plate, exposing the reactive surfaces to the aqueous solution (Supplementary Fig. 1). Water oxidation reactions were then initiated by applying a potential of +1.0 V through a Cu wire glued to the underside of these working electrodes. Oxygen bubbles with diameters in the ~144–460 μm range appeared after ~40 s of reaction time and remained pinned on the working electrodes for several hours.

### Electrochemical measurements of single bubbles

EIS data of single bubbles pinned on hematite were collected in aqueous solutions of 1 mM LiCl (5 bubbles, 25 spectra), NaCl (12 bubbles, 91 spectra), KCl (8 bubbles, 106 spectra) and 0.1 mM HCl+1 mM NaCl (4 bubbles, 31 spectra). Those on gold were collected in 1 mM NaCl (10 bubbles, 157 spectra). A low ionic strength was required to limit contributions from solution conductivity. The spectra were acquired using an ultramicroelectrode (UME; 25 µm wide Pt core of *RG* = 10 by Cromsol, U-23/25) as a working electrode, a Pt counter electrode, and a Ag wire pseudo-reference electrode. Measurements were made using a alternating current scanning electrochemical microscopy (AC-SECM) workstation (M470 BioLogic AC-SECM) equipped with a μTriCell^TM^ (Supplementary Fig. 1). The microelectrode was previously hand polished with emery paper (grade 4000) and sonicated in propanol, then ethanol and water to remove organic residues. Using a piezo-actuated stage, the UME was positioned directly over a single bubble, and lowered until the bubble was partially squeezed. This ensured an electrochemical contact between the UME, the bubble and the supporting substrate (hematite, gold, or PTFE). EIS measurements were then performed at the open circuit potential of the system by applying an alternating current (AC) of ±50 mV at 51 frequencies (*ω*) in the 1−10^5^ Hz range, using a sweep frequency protocol. The applied AC kept the system within the stability field of water. To ensure full electrochemical contact, the UME was occasionally lowered onto the bubble by 10–50 μm when needed. Using a video microscope, we monitored the apparent width of the bubbles squeezed under the UME. From these measurements, we estimate an average reduction of 3.2 ± 1.9% in diameter after each EIS measurement (Supplementary Fig. 2). This reduction is likely attributed to the loss of gases from the bubble exposed to the applied AC.

In an additional set of experiments, repeated EIS experiments of selected bubbles generated on hematite were compared to those collected after laterally moving these bubbles by 3–5 mm onto the neighboring PTFE body. Finally, EIS data were also collected on selected bubbles exposed to an externally applied potential of −1.0 V and +1.0 V. This potential was applied through the underside supporting hematite or gold electrode through a Cu wire, again using the 3-electrode set-up.

### Impedance modeling

Equivalent circuit fitting of the EIS data was performed using a Matlab (The Mathworks) code developed in our group^37,38^. The program determines the best-fitting combination of electrochemical parameters that can account for the frequency dependence of the complex impedance of the system. The model of Fig. 2d was chosen from a search by systematically increasing the level of complexity of circuit components, and evaluating the statistical significance of improvement of the fit of the model to the data for each successive combination sets. While more complex models did improve the fit to the data, they were discarded as the additional parameters became more intercorrelated.

The complex-impedance plane data (*Z*_tot_) were represented by the impedance of the UME (Z_UME_) and of the bubble (*Z*_bubble_) with:

1 $$Z_{tot} = Z_{UME} + Z_{bubble}$$

The impedance of the solution was sufficiently small that it was removed from this expression.

The *Z*_UME_ term was only active in the low frequency region, and expressed in terms of the capacitance of the UME:

2 $$Z_{{\mathrm{UME}}} = C_{{\mathrm{UME}}}^{ - 1}$$

as the resistance term of the Pt core is negligible^49,50^. Here we also assume that contributions of the electrode area that are not covered by the glass shield of the UME are negligible^50^.

The *Z*_bubble_ term pertains to the AC-induced response of ions near bubble surfaces. It is manifested in the intermediate-to-high frequency region using a bubble polarization resistance (*R*_bubble_) and double layer capacitance (*C*_bubble_)^41,51^ whereby:

3 $$Z_{{\mathrm{bubble}}}{\mathrm{ = }}\frac{{R_{bubble}}}{{{\mathrm{1 + }}({\mathrm{j}}\omega )^\varphi C_{{\mathrm{bubble}}} \cdot R_{bubble}}}$$

Here $j = \sqrt { - 1}$ is the unit imaginary number, and *ω* is the angular frequency. To investigate the possibility for heterogeneity in the system, the *φ* parameter of this constant phase element (CPE) was co-optimized within the *φ* = 0.5–1.0 range. Values of *φ* < 1.0 effectively broaden impedance value over frequency.

## Supplementary information

Supplementary Information

Peer Review File

## Data Availability

The Supplementary Information contains Supplementary Figs. 1–7 containing: a schematic representation of the experimental set-up (Supplementary Fig. 2), bubble width distribution (Supplementary Fig. 2), equivalent circuit modeling parameters for hematite (Supplementary Figs. 3–4) and gold (Supplementary Fig. 5), and additional impedance data on hematite, gold and PTFE (Supplementary Figs. 6–7). Any relevant data are available from the authors upon reasonable request.

## References

[1] Alheshibri M, Qian J, Jehannin M, Craig VSJ (2016). A history of nanobubbles. Langmuir.

[2] Zhao X, Ren H, Luo L (2019). Gas bubbles in electrochemical gas evolution reactions. Langmuir.

[3] Lauterborn W, Kurz T (2010). Physics of bubble oscillations. Rep. Prog. Phys..

[4] Zhu J (2016). Cleaning with bulk nanobubbles. Langmuir.

[5] Dai ZF, Fornasiero D, Ralston J (1999). Particle-bubble attachment in mineral flotation. J. Colloid Interface Sci..

[6] Metz T, Paust N, Zengerle R, Koltay P (2010). Capillary driven movement of gas bubbles in tapered structures. Microfluid. Nanofluid..

[7] Pereiro I, Fomitcheva Khartchenko A, Petrini L, Kaigala GV (2019). Nip the bubble in the bud: a guide to avoid gas nucleation in microfluidics. Lab Chip.

[8] Feliu S, Garcia-Galvan FR, Llorente I, Diaz L, Simancas J (2017). Influence of hydrogen bubbles adhering to the exposed surface on the corrosion rate of magnesium alloys AZ31 and AZ61 in sodium chloride solution. Mater. Corros..

[9] Huet F, Musiani M, Nogueira RP (2004). Oxygen evolution on electrodes of different roughness: an electrochemical noise study. J. Sol. State Electrochem..

[10] Chen XX (2014). Local visualization of catalytic activity at gas evolving electrodes using frequency-dependent scanning electrochemical microscopy. Chem. Comm..

[11] Trasatti S (2000). Electrocatalysis: understanding the success of DSA (R). Electrochim. Acta.

[12] Dukovic J, Tobias CW (1987). The influence of attached bubbles on potential drop and current distribution at gas-evolving electrodes. J. Electrochem. Soc..

[13] Yoon RH, Yordan JL (1986). Zeta-potential measurements on microbubbles generated using surfactances. J. Colloid Interface Sci..

[14] Leroy P, Jougnot D, Revil A, Lassin A, Azaroual M (2012). A double layer model of the gas bubble/water interface. J. Colloid Interface Sci..

[15] Graciaa A, Morel G, Saulner P, Lachaise J, Schechter RS (1995). The zeta-potential of gas-bubbles. J. Colloid Interface Sci..

[16] Jia WH, Ren SL, Hu B (2013). Effect of water chemistry on zeta potential of air bubbles. Int. J. Electrochem. Sci..

[17] Takahashi M (2005). xi potential of microbubbles in aqueous solutions: electrical properties of the gas-water interface. J. Phys. Chem. B.

[18] Beattie JK, Djerdjev AN, Warr GG (2009). The surface of neat water is basic. Farad. Disc..

[19] Healy TW, Fuerstenau DW (2007). The isoelectric point/point-of zero-charge of interfaces formed by aqueous solutions and nonpolar solids, liquids, and gases. J. Colloid Interface Sci..

[20] Lützenkirchen J, Preočanin T, Kallay N (2008). A macroscopic water structure based model for describing charging phenomena at inert hydrophobic surfaces in aqueous electrolyte solutions. Phys. Chem. Chem. Phys..

[21] Zangi R, Engberts JBFN (2005). Physisorption of hydroxide Ions from aqueous solution to a hydrophobic surface. J. Am. Chem. Soc..

[22] Preočanin T, Šupljika F, Lovrak M, Barun J, Kallay N (2014). Bubbling potential as a measure of the charge of gas bubbles in aqueous environment. Coll. Sur. A.

[23] Gabrielli C, Huet F, Nogueira RP (2005). Fluctuations of concentration overpotential generated at gas-evolving electrodes. Electrochim. Acta.

[24] Hine F, Murakami K (1980). Bubble effects on the solution IR drop in a vertical electrolizer under free and forced convection. J. Electrochem. Soc..

[25] Wang MY, Wang Z, Gong XZ, Guo ZC (2014). The intensification technologies to water electrolysis for hydrogen production - a review. Renew. Sust. Energ. Rev..

[26] Zhang LJ (2006). Electrochemically controlled formation and growth of hydrogen nanobubbles. Langmuir.

[27] Tabor RF, Grieser F, Dagastine RR, Chan DYC (2012). Measurement and analysis of forces in bubble and droplet systems using AFM. J. Colloid Interface Sci..

[28] Zeradjanin AR, Ventosa E, Masa J, Schuhmann W (2018). Utilization of the catalyst layer of dimensionally stable anodes. Part 2: Impact of spatial current distribution on electrocatalytic performance. J. Electroanal. Chem..

[29] Rincon RA (2015). Using cavity microelectrodes for electrochemical noise studies of oxygen-evolving catalysts. Chemsuschem.

[30] Battistel A, Dennison CR, Lesch A, Girault HH (2019). Local study on hydrogen and hydrogen gas bubble formation on a platinum electrode. J. Phys. Chem. C..

[31] Jiang CR, Moniz SJA, Wang AQ, Zhang T, Tang JW (2017). Photoelectrochemical devices for solar water splitting - materials and challenges. Chem. Soc. Rev..

[32] Shen SH, Lindley SA, Chen XY, Zhang JZ (2016). Hematite heterostructures for photoelectrochemical water splitting: rational materials design and charge carrier dynamics. En. Environ. Sci..

[33] Eggleston CM, Stern JR, Strellis TM, Parkinson BA (2012). A natural photoelectrochemical cell for water splitting: Implications for early Earth and Mars. Am. Miner..

[34] Doane TA (2017). A survey of photogeochemistry. Geochemical Trans..

[35] Lu AH (2019). Photoelectric conversion on Earth’s surface via widespread Fe- and Mn-mineral coatings. Proc. Natl Acad. Sci..

[36] Bard, A. J. & Mirkin, M. *Scanning Electrochemical Microscopy*. (CRC Press, 2012).

[37] Lucas M, Boily JF (2017). Electrochemical response of bound electrolyte ions at oriented hematite surfaces: a local electrochemical impedance spectroscopy study. J. Phys. Chem. C..

[38] Lucas M, Boily JF (2015). Mapping electrochemical heterogeneity at iron oxide surfaces: a local electrochemical impedance study. Langmuir.

[39] Shimizu K, Boily JF (2015). Electrochemical signatures of crystallographic orientation and counterion binding at the hematite/water interface. J. Phys. Chem. C..

[40] Shimizu K, Lasia A, Boily JF (2012). Electrochemical impedance study of the hematite/water interface. Langmuir.

[41] Macdonald, J. R. *Impedance spectroscopy: emphasizing solid materials and systems* (Wiley, 1987).

[42] Jones SF, Evans GM, Galvin KP (1999). Bubble nucleation from gas cavities - a review. Adv. Colloid Interface Sci..

[43] Shimizu K, Shchukarev A, Kozin PA, Boily JF (2012). X-ray photoelectron spectroscopy of fast-frozen hematite colloids in aqueous solutions. 4. Coexistence of alkali metal (Na+, K+, Rb+, Cs+) and chloride ions. Surf. Sci..

[44] Amos RT, Mayer KU (2006). Investigating the role of gas bubble formation and entrapment in contaminated aquifers: reactive transport modelling. J. Contam. Hydrol..

[45] Santos IR, Eyre BD, Huettel M (2012). The driving forces of porewater and groundwater flow in permeable coastal sediments: a review. Estuar. Coast. Shelf Sci..

[46] Worner M (2012). Numerical modeling of multiphase flows in microfluidics and micro process engineering: a review of methods and applications. Microfluid. Nanofluid..

[47] He C, Bi XT, Grace JR (2015). Simultaneous measurements of particle charge density and bubble properties in gas-solid fluidized beds by dual-tip electrostatic probes. Chem. Eng. Sci..

[48] Minguzzi A, Fan FRF, Vertova A, Rondinini S, Bard AJ (2012). Dynamic potential-pH diagrams application to electrocatalysts for water oxidation. Chem. Sci..

[49] Bandarenka AS, Eckhard K, Maljush A, Schuhmann W (2013). Localized electrochemical impedance spectroscopy: visualization of spatial distributions of the key parameters describing solid/liquid interfaces. Anal. Chem..

[50] Eckhard, K. & Schuhmann W. Alternating current techniques in scanning electrochemical microscopy (AC-SECM). *Analyst* **133**, 1486–1497 (2008). 10.1039/b806721j 18936824

[51] Orazem ME, Shukla P, Membrino MA (2002). Extension of the measurement model approach for deconvolution of underlying distributions for impedance measurements. Electrochim. Acta.

