# Supplementary material for: A gateway for ion transport on gas bubbles pinned onto solids
Source: Commun Chem. 2021 Mar 25;4:43. doi: 10.1038/s42004-021-00481-7 (PMC9814891; doi:10.1038/s42004-021-00481-7)
Supplement: Supplementary file 1 — Supplementary Information [file 42004_2021_481_MOESM1_ESM.docx]

**SUPPLEMENTARY INFORMATION**

**A Gateway for Ion Transport on Gas Bubbles Pinned onto Solids**

Veton Haziri^1^, Tu Pham Tran Nha ^2^, Avni Berisha^1^, Jean-François Boily^2.*^

1. *Department of Chemistry, University of Prishtina, 10 000 Prishtina, Kosovo*

2. Department of Chemistry, Umeå University, SE-901 87 Umeå, Sweden

**Table of Contents**

[Supplementary Figure 1 2](#_Toc65058562)

[Supplementary Figure 2 2](#_Toc65058563)

[Supplementary Figure 3 3](#_Toc65058564)

[Supplementary Figure 4 3](#_Toc65058565)

[Supplementary Figure 5 4](#_Toc65058566)

[Supplementary Figure 6 4](#_Toc65058567)

[Supplementary Figure 7 5](#_Toc65058568)

**
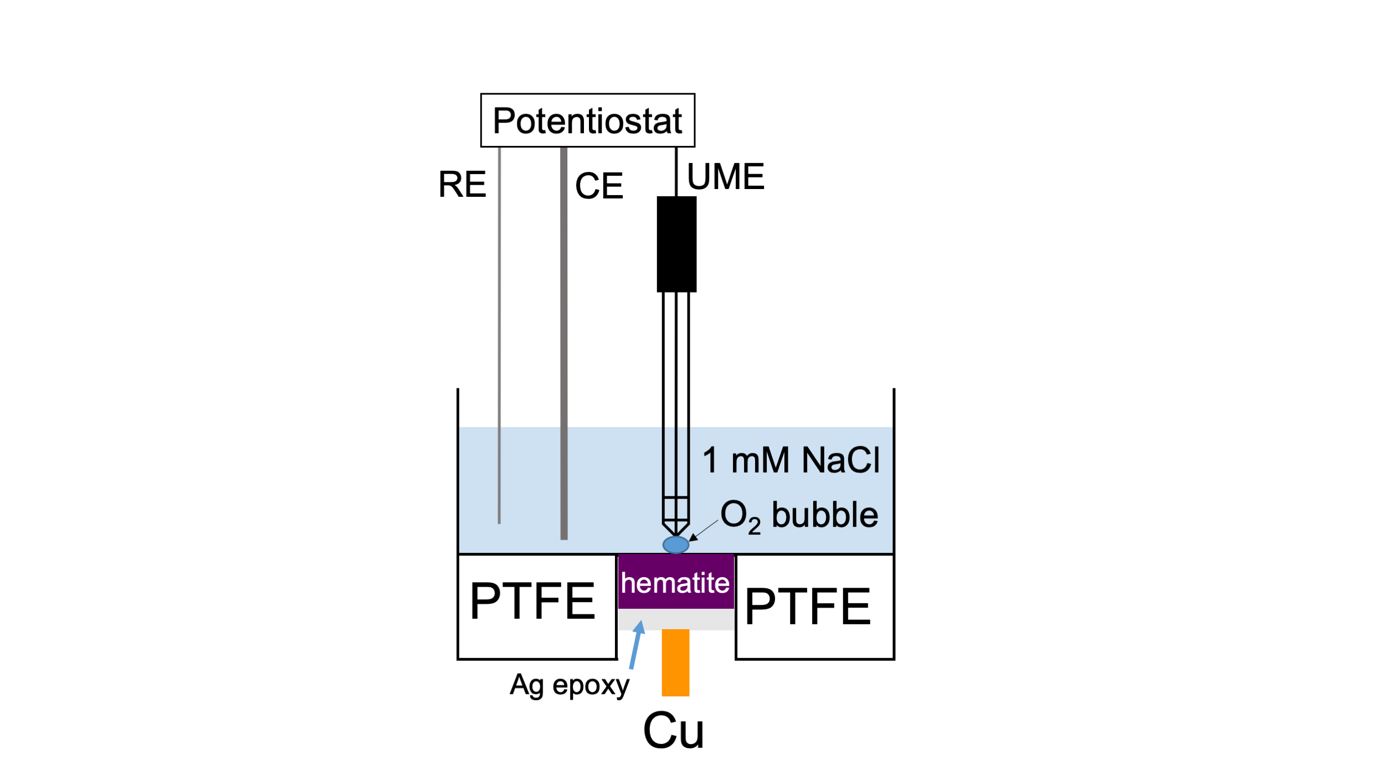
**Supplementary Figure 1 Schematic representation of experimental set-up for impedance data collection of a single bubble pinned on a hematite (‘substrate’). The bubble was produced by water oxidation directly on hematite, and remained pinned for several hours. UME=ultramicroelectrode, RE=pseudo-reference electrode (silver wire), CE=counterelectrode (Pt).


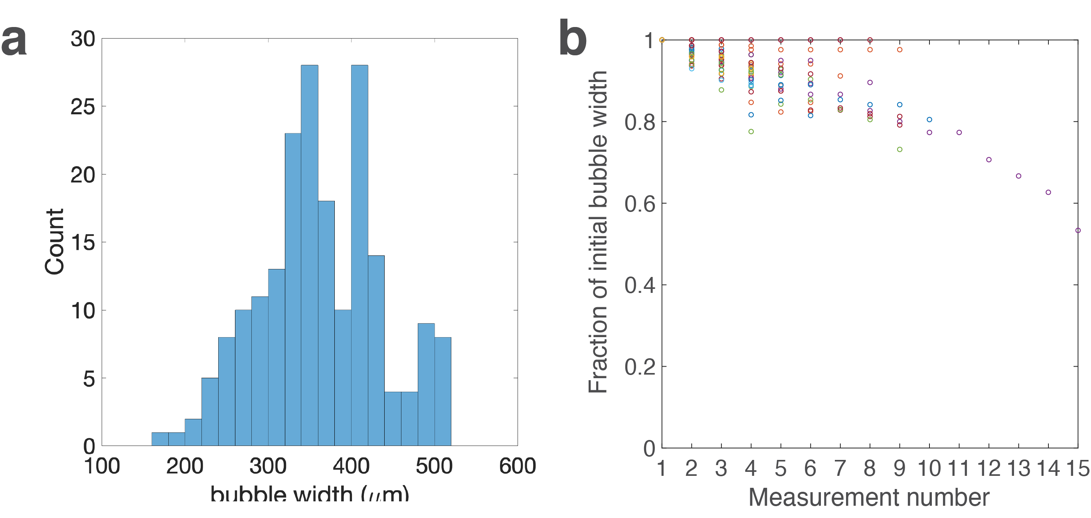


Supplementary Figure 2 Width of bubbles on pinned hematite. (a) Width distribution of all bubbles for which EIS measurements were made. (b) Fraction of initial bubble width prior each sequential EIS measurement on a single bubble (one color each). From the slopes of each data series in (b), we estimate a reduction of 3.2 ± 1.9 % in diameter after each EIS measurement.

**
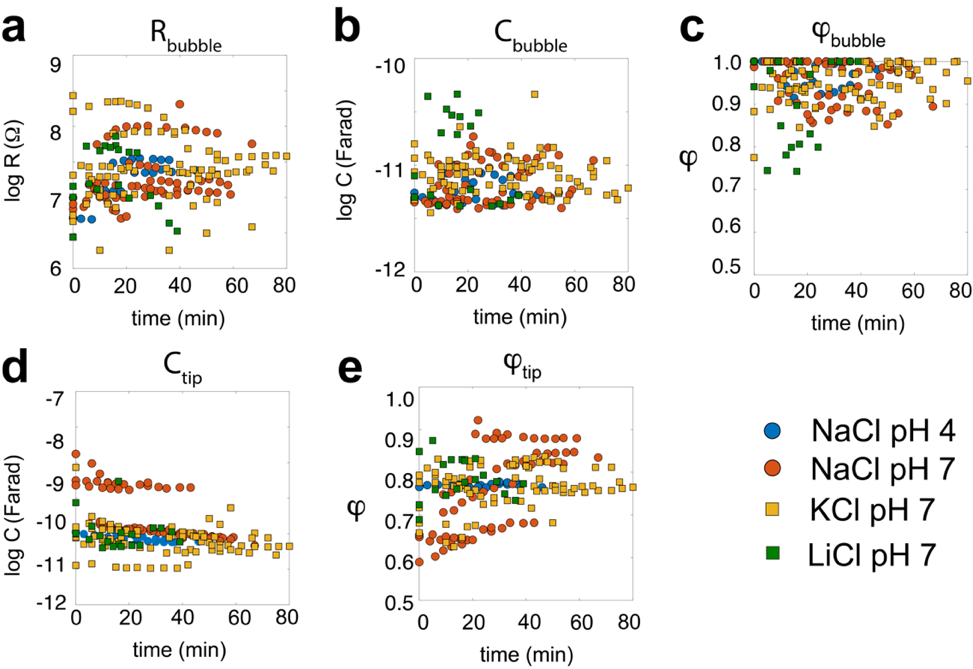
**

**
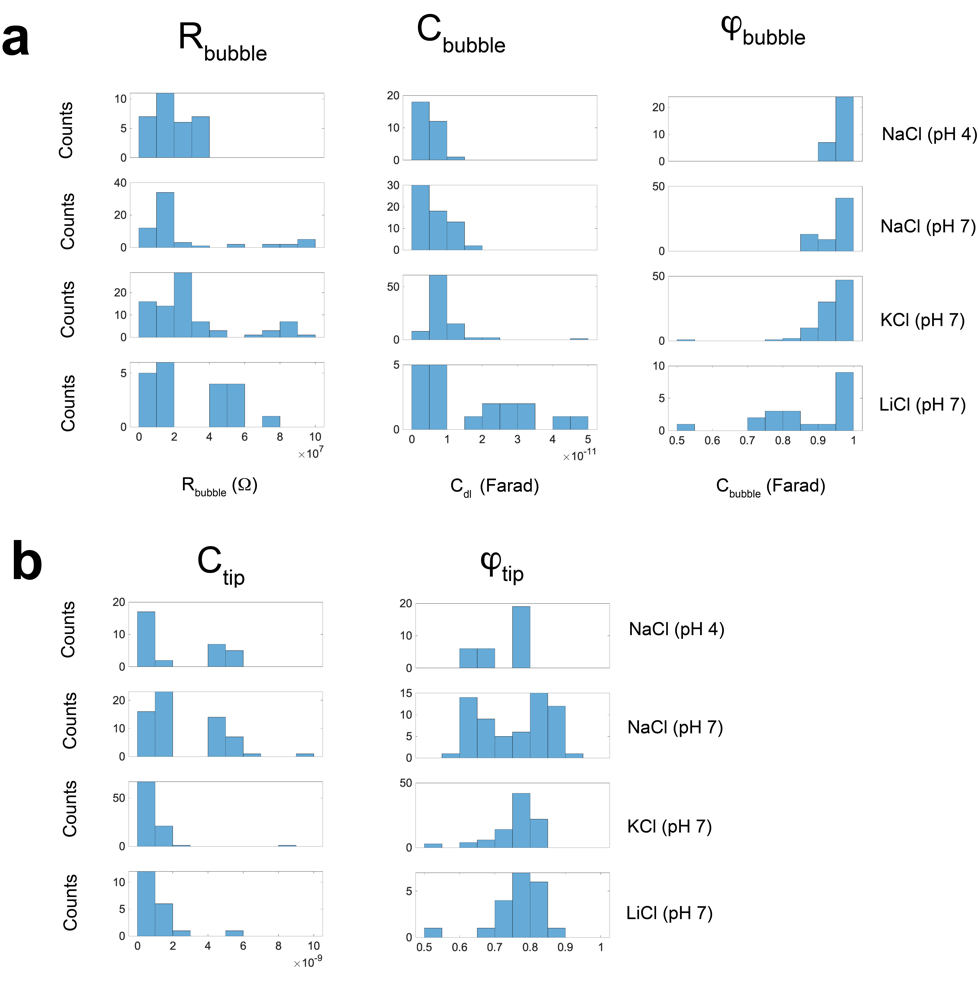
**Supplementary Figure 3 Equivalent circuit parameters for time-resolved measurements of single O_2_ bubbles pinned on hematite in 1 mM of dissolved salts.

Supplementary Figure 4 Distributions of equivalent circuit parameters for time-resolved measurements of single O_2_ bubbles pinned on hematite in 1 mM of dissolve salts, taken from Fig. S3.


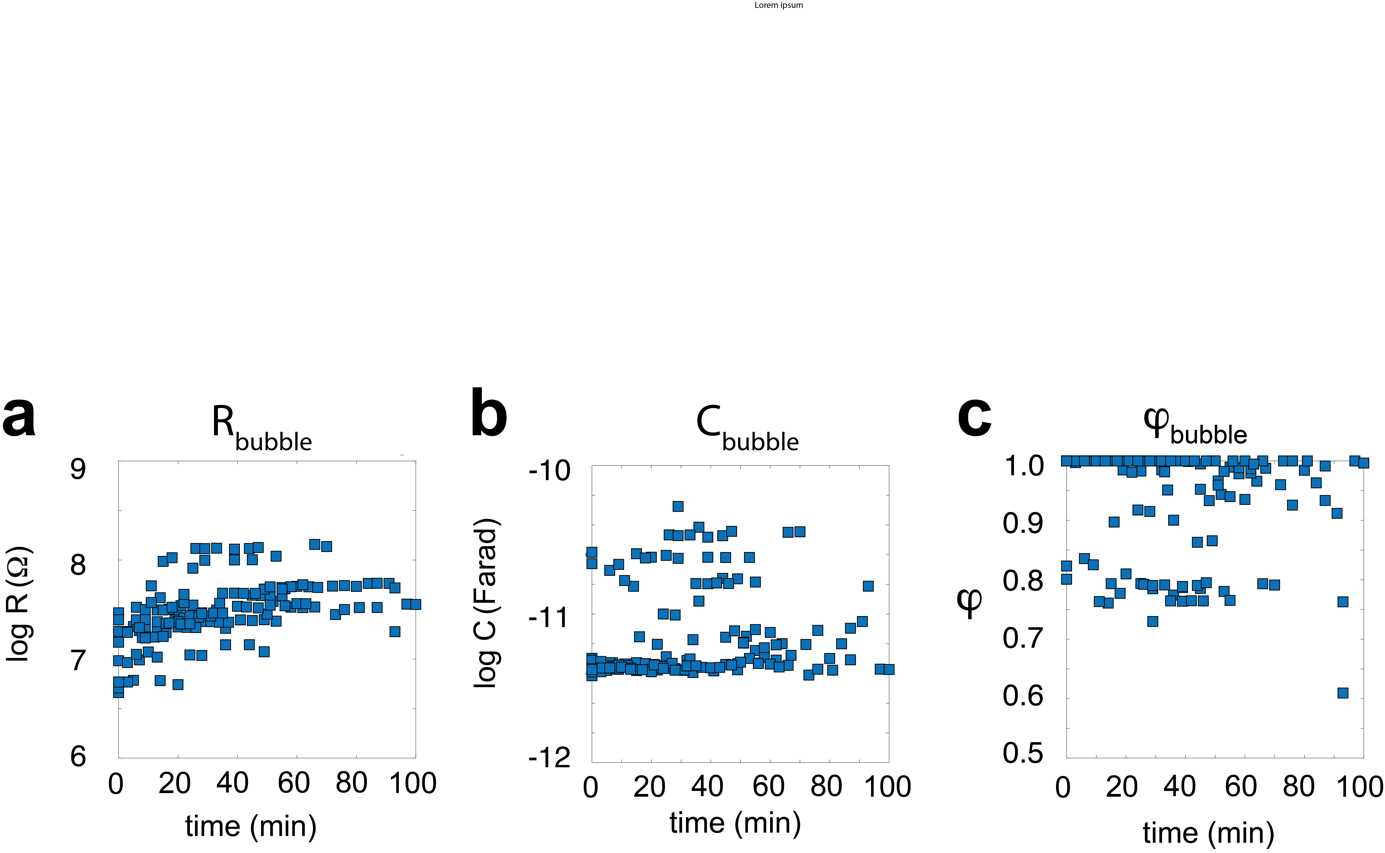


Supplementary Figure 5 Equivalent circuit parameters for time-resolved measurements of single O_2_ bubbles pinned on gold in 1 mM NaCl.


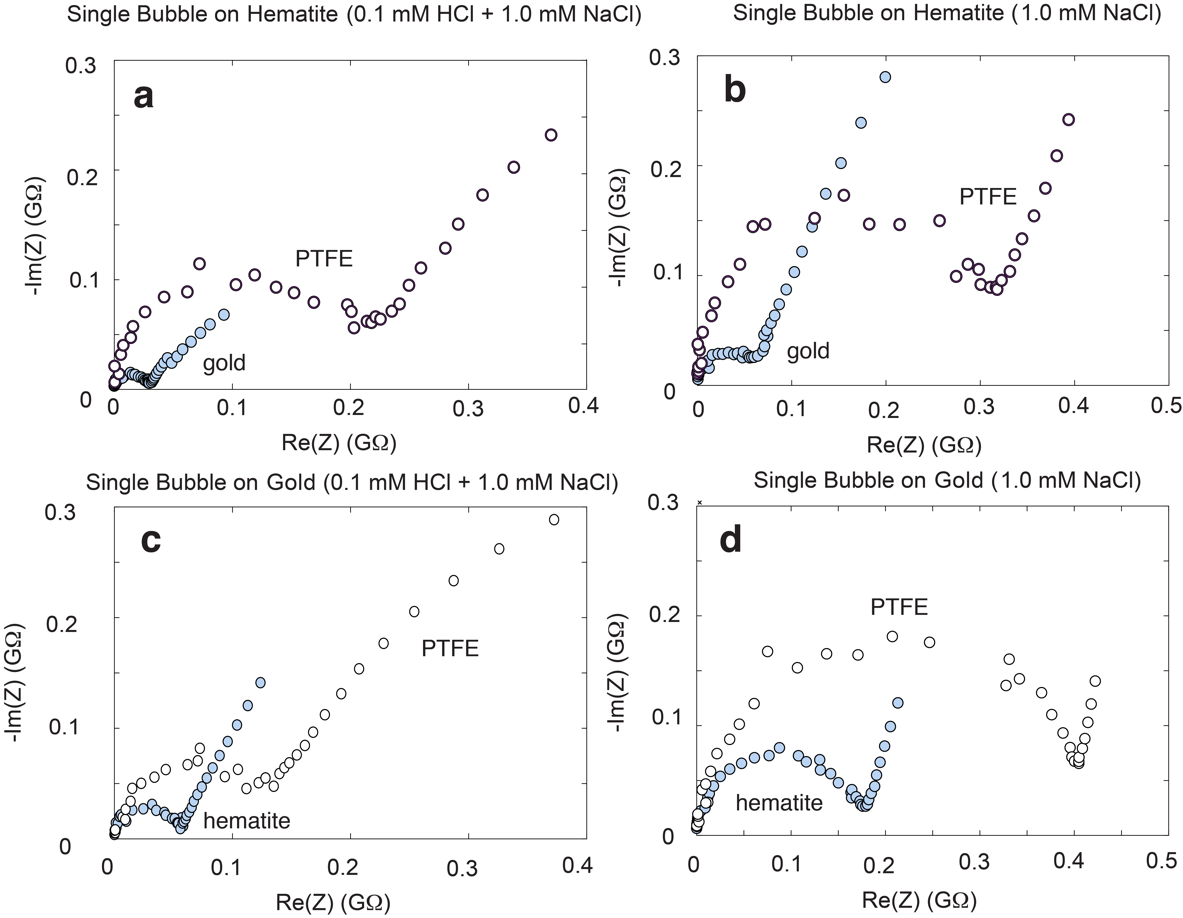


Supplementary Figure 6 Nyquist plot showing complex impedance plane data of the same single oxygen bubble first acquire on hematite or gold, then on PTFE. The bubble became more resisting on PTFE. EIS were collected in the 1-10^5^ Hz range in 1.0 mM NaCl at 25 °C.

**
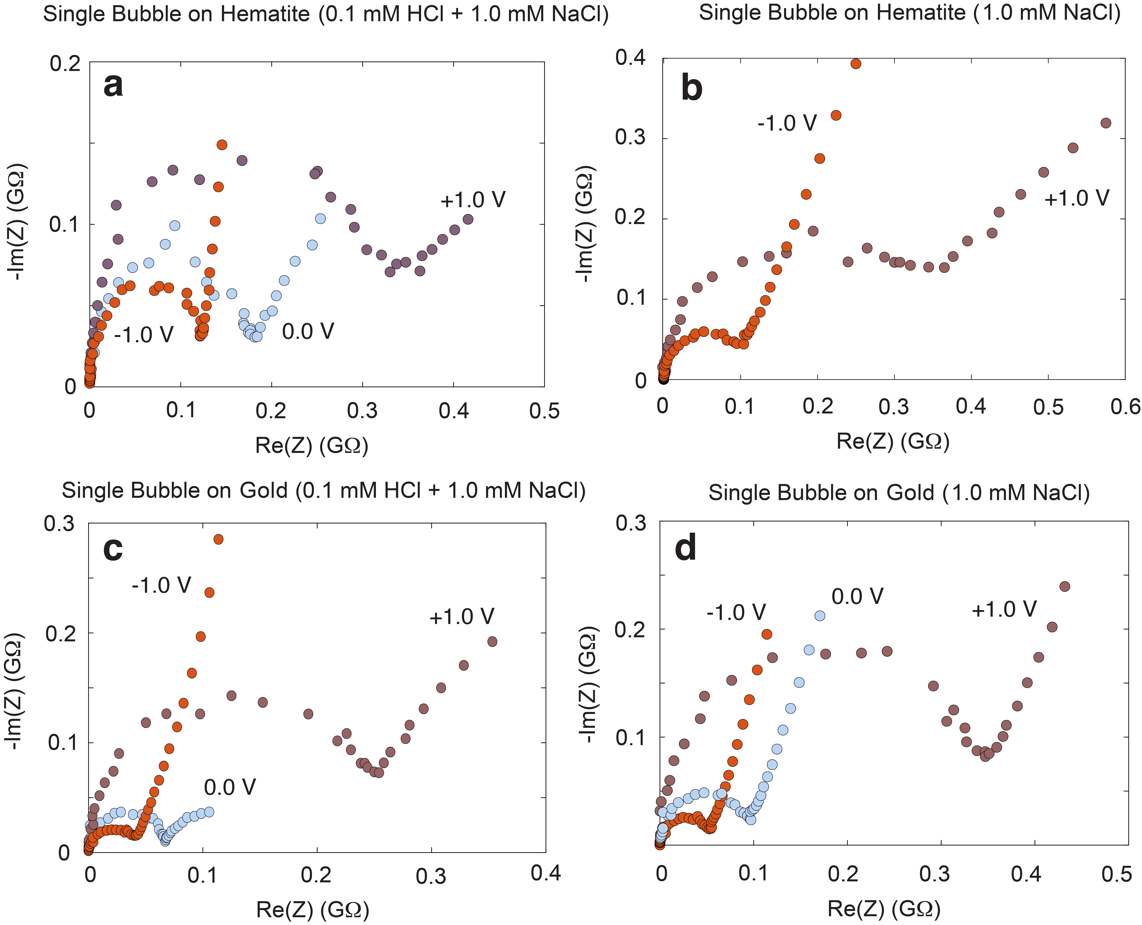
**

Supplementary Figure 7 Nyquist plot showing the complex-plane impedance data of a single bubble on (a-b) hematite and (c-d) gold under applied potentials of -1.0, 0.0 and +1.0 V. EIS were collected in the 1-10^5^ Hz range in 1.0 mM NaCl at 25 °C. Solid lines are fits from the equivalent circuit model of Fig. 2a.
