# Supplementary material for: A gateway for ion transport on gas bubbles pinned onto solids
Source: Commun Chem. 2021 Mar 25;4:43. doi: 10.1038/s42004-021-00481-7 (PMC9814891; doi:10.1038/s42004-021-00481-7)
Supplement: Supplementary file 2 — Peer Review File [file 42004_2021_481_MOESM2_ESM.pdf]

Reviewers' comments:

Reviewer #1 (Remarks to the Author):

The authors study oxygen bubbles pinned onto hematite or gold surfaces and analyse the charge transfer in between. Main achievement of the manuscript consists in the detection that the overlapping electric double layers of gas bubbles and the semi-conductive substrate form a gateway for the transfer of counter ions.

This is definitely a valuable result with significant relevance to other branches like froth flotation or catalysis.
I recommend a publication of the manuscript if the following issues have been adequately addressed:

- More details on the electrolyte preparation should be given. Is it exposed to /saturated with air? Might adsorption and gas flux (e.g. N2) across the interface caused by diffusion affect the results?

- Is the size of the bubbles really constant over time? The wetting details at the UME will have a strong impact! (see e.g. the current transient for the H2 bubble evolution at microelectrodes in our papers)

- The reasons behind the considerable scatter of the data in Fig.2 e-f hould be discussed a bit more detailed.

- To what extent might the different wetting behavior at hematite and PTFE affect the results?

- Iron oxides are known to be used as catalysts for OER in alkaline electrolytes (e.g. Doyle, Phys. Chem. Chem. Phys., 2013, 15, 5224). Please relate yor results obtained at different voltages to what is already known.

- It would be helpful to discuss also the frequency dependence (Bode plots) in Figs 3 and 4. This would allow to check what lateral dimension of the hematite EDL is involved in the effect observed.

- Fig. 3b: Different symbols in the plot remained unexplained.

- Line 172 “To test for the heterogeneity of the electrochemical response…” – It remains unclear what is meant with heterogeneity?

- Bibliography needs to be improved: typos and missing publishing year everywhere

Reviewer #2 (Remarks to the Author):

The manuscript A Gateway for Ion Transport on Gas Bubbles Pinned onto Solids considers an interesting process of interaction of the gas (oxygen) bubbles and the charged solid surface (hematite). The idea is novel and it is expected to be interesting to the scientific community. However, despite the novelty, the article has imperfections that should be eliminated before publishing.

From the electrochemical Impedance Spectroscopy (EIS) measurements and measurements with hematite single crystal electrode authors conclude that gateway for ion transport is established when overlapping EDLs of bubbles and solid surfaces are of the same polarity. This conclusion is partly supported by measurements but a clearer interpretation of the results and additional measurements is lacking:

(i) Measurements were made at pH 4 and pH 7 and in Li, Na and K chloride aqueous solutions.

At pH = 4 the hematite surface is positively charged while gas bubble is near point of zero charge, that is uncharged.

At pH = 7 the hematite is near the point of zero charge (or slightly positively charged) while gas bubble is negatively charged.

The results for these two cases are expected to be different. An analysis of these results is lacking.

(ii) The effect of different cations was not observed. Despite the different sizes of these ions, their hydrodynamic radii and molar conductivity. How authors explain this?

(ii) it will be interesting to perform measurements at different salt concentrations and examine the effect of ionic strength

Reading the article raises additional questions:

(iv) a bubble formed on the surface of the hematite covers the surface of the oxide. What happens to the EIL in that part of the surface?

(v) in the introduction part author define the surface groups (OH) on the surface of gas bubble. What is the physical meaning of such a model? A better explanation of EIL around bubbles would be the distribution of OH^- and H^+ ions between the interface and the bulk of the solution and the different affinity of OH^- and H^+ ions to the surface of the bubble.

(vi) oxygen bubbles were electrochemically produced by water oxidation at hematite surfaces. Whether oxidation / reduction processes also affect the chemical composition of hematite surface? are the surface properties of hematite known after possible surface transformations?

(vii) in the introduction parts it is written that "solid surfaces can produce gas bubbles". This is conceptually wrong. Oxygen bubbles are formed by the electrolysis of water.

(vii) The meaning of the last sentence in the third paragraph (page 3, lines 122-124) is not clear.

(viii) The meaning of the first sentence in the fourth paragraph (page 3, lines 126-127) is not clear. It seems that one "of" is redundant.

(ix) Figure 1a. If the pH of the solution is about 7, both H^+ and OH^- ions are present. There are OH^- and Na^+ ions in the EIL, but H^+ and Cl^- ions are also present. The equilibrium state in EIL is also achieved. Above all, the special attention should be paid to the electro neutrality condition.

According to above I recommend this paper for publication after major revisions.

The manuscript *A Gateway for Ion Transport on Gas Bubbles Pinned onto Solids* considers an interesting process of interaction of the gas (oxygen) bubbles and the charged solid surface (hematite). The idea is novel and it is expected to be interesting to the scientific community. However, despite the novelty, the article has imperfections that should be eliminated before publishing.

From the electrochemical Impedance Spectroscopy (EIS) measurements and measurements with hematite single crystal electrode authors conclude that gateway for ion transport is established when overlapping EDLs of bubbles and solid surfaces are of the same polarity. This conclusion is partly supported by measurements but a clearer interpretation of the results and additional measurements is lacking:

(i) Measurements were made at pH 4 and pH 7 and in Li, Na and K chloride aqueous solutions.

At pH = 4 the hematite surface is positively charged while gas bubble is near point of zero charge, that is uncharged.

At pH = 7 the hematite is near the point of zero charge (or slightly positively charged) while gas bubble is negatively charged.

The results for these two cases are expected to be different. An analysis of these results is lacking.

(ii) The effect of different cations was not observed. Despite the different sizes of these ions, their hydrodynamic radii and molar conductivity. How authors explain this?

(ii) it will be interesting to perform measurements at different salt concentrations and examine the effect of ionic strength

Reading the article raises additional questions:

(iv) a bubble formed on the surface of the hematite covers the surface of the oxide. What happens to the EIL in that part of the surface?

(v) in the introduction part author define the surface groups (OH) on the surface of gas bubble. What is the physical meaning of such a model? A better explanation of EIL around bubbles would be the distribution of OH^- and H^+ ions between the interface and the bulk of the solution and the different affinity of OH^- and H^+ ions to the surface of the bubble.

(vi) oxygen bubbles were electrochemically produced by water oxidation at hematite surfaces. Whether oxidation / reduction processes also affect the chemical composition of hematite surface? are the surface properties of hematite known after possible surface transformations?

(vii) in the introduction parts it is written that “solid surfaces can produce gas bubbles”. This is conceptually wrong. Oxygen bubbles are formed by the electrolysis of water.

(vii) The meaning of the last sentence in the third paragraph (page 3, lines 122-124) is not clear.

(viii) The meaning of the first sentence in the fourth paragraph (page 3, lines 126-127) is not clear. It seems that one “of” is redundant.

(ix) Figure 1a. If the pH of the solution is about 7, both H^+ and OH^- ions are present. There are OH^- and Na^+ ions in the EIL, but H^+ and Cl^- ions are also present. The equilibrium state in EIL is also achieved. Above all, the special attention should be paid to the electro neutrality condition.

<u>According to above I recommend this paper for publication after major revisions.</u>

Authors' responses (in blue) to Reviewers' comments (in red)

Reviewer #1 (Remarks to the Author):

The authors study oxygen bubbles pinned onto hematite or gold surfaces and analyse the charge transfer in between. Main achievement of the manuscript consists in the detection that the overlapping electric double layers of gas bubbles and the semi-conductive substrate form a gateway for the transfer of counter ions.

This is definitely a valuable result with significant relevance to other branches like froth flotation or catalysis. I recommend a publication of the manuscript if the following issues have been adequately addressed:

First, we wish to thank Reviewer #1 for a highly insightful report of our work. These comments have certainly improved the clarity of our message.

- More details on the electrolyte preparation should be given. Is it exposed to /saturated with air? Might adsorption and gas flux (e.g. N2) across the interface caused by diffusion affect the results?

Aqueous solutions of 1.0 mM LiCl, NaCl, or KCl and in a mixed 1.0 mM NaCl/0.1 mM HCl solutions were prepared using ultrapure water (18.2 MΩ). The temperature of the lab was 25 ^{o}C all time. The air-saturated solutions were not saturated with inert gas (like N_2) as they were ultimately saturated with dissolved oxygen from the gas bubble formation step prior the EIS measurements.

In the revised manuscript we now write on lines 254-255:

"Oxidation reactions were carried out in aqueous solutions of air-saturated 1.0 mM LiCl, NaCl or KCl and in a mixed 1.0 mM NaCl/0.1 mM HCl solution. The solutions then became oxygen-saturated after electrochemical bubble production. "

- Is the size of the bubbles really constant over time? The wetting details at the UME will have a strong impact! (see e.g. the current transient for the H2 bubble evolution at microelectrodes in our papers)

This is certainly a good point. We noticed that the bubbles did have, occasionally, a tendency to shrink with time, perhaps due to the disequilibrium induced by the applied AC current. After a while (after achieving the equilibrium), however, the bubble diameter was stable and did not change anymore. Most importantly for this question, we did monitor bubble diameter throughout the course of all experiments to see whether there would be any correlation between size and the electrochemical response. No correlation was however found.

To address this comment in the revised manuscript we wrote (lines 281-282):

While we did find that the bubbles occasionally shrank during the first 1-5 EIS measurements, the electrochemical parameters were not affected.

- The reasons behind the considerable scatter of the data in Fig.2 e-f hould be discussed a bit more detailed.

This scatter might have been especially caused by the quality of the electrochemical contact between the UME and the bubble. Ensuring this contact was one of the greatest experimental challenges of this work. This is why we report many trials for each conditions, and also report our parameters in the histograms of the SI.

- To what extent might the different wetting behavior at hematite and PTFE affect the results?

Our choice of a hydrophobic PTFE surface was motivated to minimize any possibility of EDL, and thus accumulation of electrolyte ions, between the bubbles and the PTFE surface. On lines 193-194 we now write:

PTFE is not only an insulator but its low wettability minimized that an EDL is formed on its surface.

- Iron oxides are known to be used as catalysts for OER in alkaline electrolytes (e.g. Doyle, Phys. Chem. Chem. Phys., 2013, 15, 5224). Please relate yor results obtained at different voltages to what is already known.

We chose to refrain from doing such a comparison because our work was focused to circumneutral and mildly acidic solutions. Voltages in alkaline solutions are different because the strongly deprotonated hematite surfaces facilitate OER.

- It would be helpful to discuss also the frequency dependence (Bode plots) in Figs 3 and 4. This would allow to check what lateral dimension of the hematite EDL is involved in the effect observed.

As they are highly comparable to those of Fig. 2, we did not find it useful to show these data along frequency.

- Fig. 3b: Different symbols in the plot remained unexplained.

They are repeated measurements on a single bubble. We now specified this in the figure caption of Fig. 3b.

- Line 172 "To test for the heterogeneity of the electrochemical response…" – It remains unclear what is meant with heterogeneity?

A few lines down we say ", and these likely reflect the homogeneous distribution of charged ions at on bubble surfaces." Technically, this heterogeneity is manifested by the breadth of the frequency-resolved distribution of the impedance data.

- Bibliography needs to be improved: typos and missing publishing year everywhere

We have now revised the bibliography for these discrepancies.

Reviewer #2 (Remarks to the Author):

The manuscript A Gateway for Ion Transport on Gas Bubbles Pinned onto Solids considers an interesting process of interaction of the gas (oxygen) bubbles and the charged solid surface (hematite). The idea is novel and it is expected to be interesting to the scientific community. However, despite the novelty, the article has imperfections that should be eliminated before publishing.

We wish to thank Reviewer #2 for these comments, and for helping us improving the clarity of our paper.

From the electrochemical Impedance Spectroscopy (EIS) measurements and measurements with hematite single crystal electrode authors conclude that gateway for ion transport is established when overlapping EDLs of bubbles and solid surfaces are of the same polarity. This conclusion is partly supported by measurements but a clearer interpretation of the results and additional measurements is lacking:

(i) Measurements were made at pH 4 and pH 7 and in Li, Na and K chloride aqueous solutions.
At pH = 4 the hematite surface is positively charged while gas bubble is near point of zero charge, that is uncharged.

At pH = 7 the hematite is near the point of zero charge (or slightly positively charged) while gas bubble is negatively charged.

The results for these two cases are expected to be different. An analysis of these results is lacking.

We originally chose these pH values precisely for these differences in surface charge. However, as we found no differences in the spread of electrochemical parameters between pH 4 and 7 (Figure S2) we decided to

avoid a discussion on the pH effect for a later study where we would explore considerably more acidic and alkaline solutions. This is why we relegated the pH 4 data to the SI for the time being.

(ii) The effect of different cations was not observed. Despite the different sizes of these ions, their hydrodynamic radii and molar conductivity. How authors explain this?

The technique is likely unresponsive to these differences, which many be hidden in the relatively large uncertainty of the measurements.

(ii) it will be interesting to perform measurements at different salt concentrations and examine the effect of ionic strength

This proves to be a difficult task for EIS, as the signal from the substrate is dampened by solutions of high ionic strengths (high conductivity).

Reading the article raises additional questions:
(iv) a bubble formed on the surface of the hematite covers the surface of the oxide. What happens to the EIL in that part of the surface?

Another good question. We also wondered whether there would be an effect, and tested this by performing an EIS experiment using hematite as the working electrode. We however found no difference between a pristine and bubble-coated hematite. We now state this on lines 181-184:

"Finally, we discard the possibility that the bubbles altered the EDL of hematite, as additional EIS measurements using hematite as a working electrode showed no differences in its electrochemical response (not shown)."

(v) in the introduction part author define the surface groups (OH) on the surface of gas bubble. What is the physical meaning of such a model? A better explanation of EIL around bubbles would be the distribution of OH– and H+ ions between the interface and the bulk of the solution and the different affinity of OH– and H+ ions to the surface of the bubble.

This model is taken from the literature (references 13-17), and is derived from negative zeta potentials of bubbles, pointing to a preferential accumulation of OH- ions.

(vi) oxygen bubbles were electrochemically produced by water oxidation at hematite surfaces. Whether oxidation / reduction processes also affect the chemical composition of hematite surface? are the surface properties of hematite known after possible surface transformations?

We did not measure this but EIS measurements of hematite as a working electrode do not show any differences.

(vii) in the introduction parts it is written that "solid surfaces can produce gas bubbles". This is conceptually wrong. Oxygen bubbles are formed by the electrolysis of water.

The processes we cite involve more than electrolytic production of water, and include accumulation of gases by cavitation. We attempted to clarify this point by writing:

"Solid surfaces can collect gas bubbles by …"

(vii) The meaning of the last sentence in the third paragraph (page 3, lines 122-124) is not clear.

We rewrote these lines:

"At the core of these phenomena lies the ability bubbles to electrochemically communicate with solid surfaces upon which they reside, and thus to exchange ions populating the EDL."

With:

"At the core of these phenomena lies the possibility for bubbles to electrochemically communicate with

solid surfaces upon which they are pinned by exchanging ions populating overlapping EDL. “

(viii) The meaning of the first sentence in the fourth paragraph (page 3, lines 126-127) is not clear. It seems that one “of” is redundant.

‘of’ is now removed. Thank you.

(ix) Figure 1a. If the pH of the solution is about 7, both H^+ and OH^- ions are present. There are OH^- and Na^+ ions in the EIL, but H^+ and Cl^- ions are also present. The equilibrium state in EIL is also achieved. Above all, the special attention should be paid to the electro neutrality condition.

This diagram emphasizes the EDL portion of the bubble, in which there is an enrichment of OH- ions at the air/water interface, which is deduced by the negative zeta potentials of bubbles. Na^+ ions, present in mM concentration, are the dominant counterions.

According to above I recommend this paper for publication after major revisions.

REVIEWERS' COMMENTS:

Reviewer #1 (Remarks to the Author):

I have examined the revision of the manuscript and agree mostly with the changes they made. I now recommend the publication of the manuscript provided that the following rather unsharp formulation of the authors, namely "While we did find that the bubbles occasionally shrank during the first 1-5 EIS measurements, the
electrochemical parameters were not affected.", is precised:

Please specify by which per cent the bubble diameter shrinks.

Reviewer #2 (Remarks to the Author):

All points raised in the previous round of review have been satisfactorily addressed.

REVIEWERS' COMMENTS:

Reviewer #1 (Remarks to the Author):

I have examined the revision of the manuscript and agree mostly with the changes they made. I now recommend the publication of the manuscript provided that the following rather unsharp formulation of the authors, namely "While we did find that the bubbles occasionally shrank during the first 1-5 EIS measurements, the electrochemical parameters were not affected.", is precised:

Please specify by which per cent the bubble diameter shrinks.

We thank Reviewer #1 for insisting on this point. We have gone back to our note and tabulated all recorded bubble dimensions between each EIS measurements. In a new Fig. S2 we now report the distribution of bubble volumes (Fig. S2a) and the change in volume (Fig. S2b in term of fraction of initial volume) in repeated measurements. We now can more confidently report that the bubbles decreased on average by 3.2 ± 1.9 % per each EIS measurement.

In the main text (lines 280-283) we also include the following lines to address the Reviewer's very pertinent question:

Using a video microscope, we monitored the apparent width of the bubbles squeezed under the UME. From these measurements, we estimate a reduction of 3.2 ± 1.9 % in diameter after each EIS measurement. This reduction is likely attributed to the loss of gases from the bubble exposed to the externally applied AC.

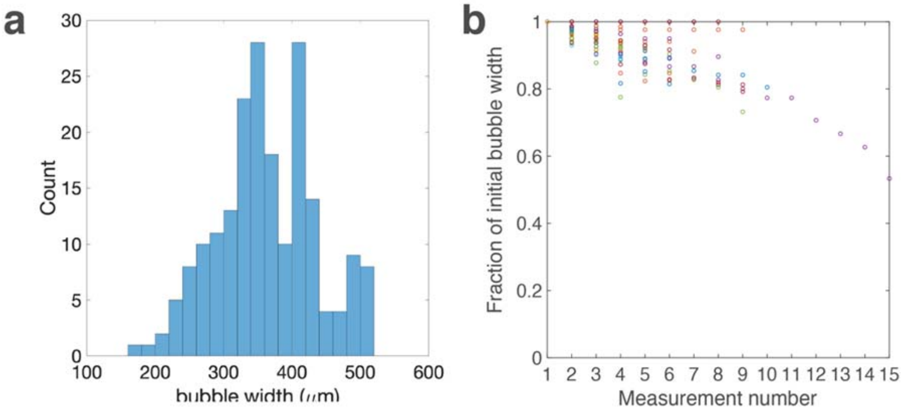

Figure S2 Width of bubbles on pinned hematite. (a) Width distribution of all bubbles for which EIS measurements were made. (b) Fraction of initial bubble width prior each sequential EIS measurement on a single bubble (one color each). From the slopes of each data series in (b), we estimate a reduction of 3.2 ± 1.9 % in diameter after each EIS measurement.

Reviewer #2 (Remarks to the Author):

All points raised in the previous round of review have been satisfactorily addressed.

Thank you.
